# Fine-Tuning Tumor Endothelial Cells to Selectively Kill Cancer

**DOI:** 10.3390/ijms18071401

**Published:** 2017-06-30

**Authors:** Emilie Uldry, Seraina Faes, Nicolas Demartines, Olivier Dormond

**Affiliations:** Department of Visceral Surgery, Lausanne University Hospital, Pavillon 4, Avenue de Beaumont, 1011 Lausanne, Switzerland; emilie.uldry@chuv.ch (E.U.); seraina.faes@chuv.ch (S.F.); demartines@chuv.ch (N.D.)

**Keywords:** cancer, endothelial cell, angiogenesis, therapies

## Abstract

Tumor endothelial cells regulate several aspects of tumor biology, from delivering oxygen and nutrients to shaping the immune response against a tumor and providing a barrier against tumor cell dissemination. Accordingly, targeting tumor endothelial cells represents an important modality in cancer therapy. Whereas initial anti-angiogenic treatments focused mainly on blocking the formation of new blood vessels in cancer, emerging strategies are specifically influencing certain aspects of tumor endothelial cells. For instance, efforts are generated to normalize tumor blood vessels in order to improve tumor perfusion and ameliorate the outcome of chemo-, radio-, and immunotherapy. In addition, treatment options that enhance the properties of tumor blood vessels that support a host’s anti-tumor immune response are being explored. Hence, upcoming anti-angiogenic strategies will shape some specific aspects of the tumor blood vessels that are no longer limited to abrogating angiogenesis. In this review, we enumerate approaches that target tumor endothelial cells to provide anti-cancer benefits and discuss their therapeutic potential.

## 1. Introduction

Endothelial cells line the inner surface of blood vessels and constitute a selective barrier between blood and tissue. Despite a very simple phenotype, endothelial cells play a critical role in a variety of physiological processes, including the maintenance of blood fluidity, the trafficking of blood cells, innate and adaptive immunity, and coagulation [1]. Accordingly, inappropriate endothelial cell responses are observed in numerous diseases, such as atherosclerosis, sepsis, allograft rejection, and cancer [2]. Therefore, therapeutic approaches that selectively target functions regulated by endothelial cells are of high interest.

**Figure 1:**
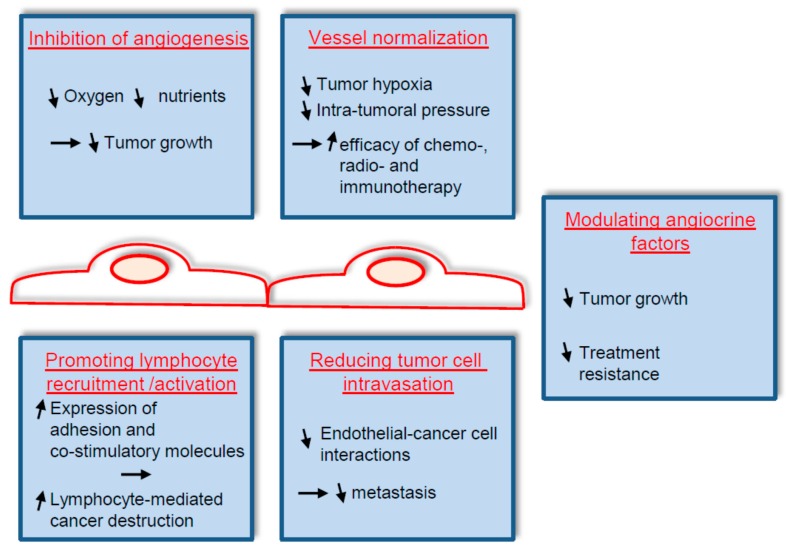
Therapeutic options that target the tumor endothelium and that will be discussed here are depicted. Inhibition of angiogenesis aims to starve tumor cells by blocking the formation of new blood vessels in the tumor. Vessel normalization leads to the formation of mature blood vessels, resulting in reduced tumor hypoxia and reduced resistance to chemo-, radio- and immunotherapy. Promoting lymphocytes’ recruitment and activation by stimulating the expression of adhesion and co-stimulatory molecules by tumor endothelial cells to enhance a host’s anti-tumor response. Reducing tumor cell invasion to prevent metastasis. Modulating angiocrine factors to reduce tumor growth and resistance to anti-cancer therapies. Rightwards arrow signify implies; Upwards arrow: increased; Downwards arrow: decreased.

The importance of endothelial cells in the context of cancer has been extensively investigated. Already in 1945, it was reported that transplanted tumors in mice are able to recruit capillaries from the host [3]. In the early 1970s, a tumor associated factor that stimulates the formation of new blood vessels in cancer was isolated, and a therapeutic intervention to block this factor was proposed [4,5]. The arising paradigm in which blocking the formation of new blood vessels in tumors would block tumor progression was the starting point of an extensive research domain that is still progressing today. During the last decades, therapies that disrupt the tumor endothelium mainly by blocking the vascular endothelial growth factor (VEGF) and its receptors have been approved for the treatment of several advanced cancers, hence validating the initial paradigm [6,7]. Nonetheless, despite very successful pre-clinical studies, anti-angiogenic treatments have provided only limited benefits in cancer patients. Resistance mechanisms to anti-VEGF therapies have been identified, such as the activation of other pro-angiogenic pathways, or the use of alternative modes of vascularization [8,9]. Whilst a part of the ongoing research focuses on circumventing these resistance mechanisms, novel therapeutic designs based on specific features of the tumor endothelium are emerging. Tumor vessels are frequently leaky, leading to high interstitial fluid pressure and reduced blood perfusion and oxygenation. The consequences of such dysfunctioning endothelium are a reduced delivery of cytotoxic agents and a resistance to radiotherapy. Hence, the normalization of tumor vessels was proposed as another strategy to modulate tumor endothelium [10,11]. In addition, tumor endothelial cells influence the host’s immune response by controlling the penetration of immune cells into the tumor and by modulating their activity. Accordingly, approaches that stimulate the recruitment and activation of lymphocytes by tumor endothelial cells are tested [12,13,14]. Thus, future therapies that target the tumor endothelium are no longer restricted to blocking angiogenesis and will be discussed here (Figure 1).

## 2. Disrupting the Formation of New Blood Vessels

As mentioned earlier, tumor endothelial cells are necessary to assure tumor blood perfusion and ensure the delivery of oxygen and nutrients as well as the removal of metabolic waste. Accordingly, tumors stimulate the formation of new blood vessels from preexisting ones in a process called sprouting angiogenesis. Besides VEGF, a plethora of factors were shown to partake in sprouting angiogenesis, including angiopoietins, basic fibroblast growth factor, integrins, and δ-like 4 ligand/NOTCH homolog 1 (NOTCH1) [15]. In addition, intracellular factors such as focal adhesion kinase or mechanistic target of rapamycin (mTOR) are important signaling intermediates in angiogenesis [16,17]. Blocking such factors is very successful in reducing tumor growth in preclinical models; tumor regression is however rarely achieved [18]. Most anti-angiogenic agents, based primarily on VEGF/VEGFR inhibition, have also failed to provide long term benefits in cancer patients, increasing their overall survival and progression free survival only by a few months [19,20]. It appears that, whilst a substantial number of tumors are intrinsically resistant to anti-VEGF therapies, others quickly escape inhibition [21]. Several studies have identified multiple mechanisms of resistance, such as the activation of other pro-angiogenic pathways, and have characterized the complex role of stromal and cancer cells in mediating resistance [8,22]. In addition, tumor vasculature is heterogeneous, where specific vessel subtypes do not respond to anti-VEGF therapies [23]. More worryingly, besides sprouting angiogenesis, tumors are able to acquire blood perfusion by other modes of vascularization [24]. For instance, cancer cells can be incorporated into blood vessel walls in a process called vascular mimicry [25]. In addition, cancer cells can co-opt an existing vasculature [26]. This demonstrates that the paradigm in which tumors develop their own blood supply in order to grow is not entirely true. Accordingly, clinical studies have demonstrated the presence of proliferating lung cancer cells in the absence of angiogenesis [27]. Hence, with different mechanisms and pro-angiogenic factors leading to tumor vascularization, the failure of anti-VEGF therapies in clinical trials is not surprising. It highlights that, as for most other targeted therapies, a single inhibition is not sufficient to provide prolonged anti-tumor efficacy. Whereas the use of biomarkers might help identify tumors that will mostly benefit from anti-angiogenic treatments [28], the chance to provide long-term anti-tumor efficacy by blocking the formation of new tumor blood vessels from pre-existing ones appears rather limited.

Most of the initial strategies to prevent tumor angiogenesis have focused on blocking growth factors or factors implicated in endothelial cell proliferation, survival, and migration. Recently, a novel approach based on targeting the endothelial metabolism rather than growth factors has been proposed [29]. Obviously, the production of biomass and energy by endothelial cells is key to angiogenesis, and therefore represents an attractive target for anti-angiogenic therapies. Sprouting angiogenesis relies heavily on glycolysis, and blocking the glycolytic activator 6-phosphofructo-2-kinase/fructose-2,6-biphosphatase 3 (PFKFB3) either genetically or chemically reduces vessel formation in both physiological and pathological angiogenesis [30,31]. In addition, the inhibition of PFKFB3 also amplifies the anti-angiogenic effects of sunitinib, a VEGF receptor tyrosine kinase inhibitor [31]. Of note, blocking PFKFB3 does not reduce tumor growth in tumor mouse models and does not affect the number of tumor blood vessels, but rather induces vessel normalization [32]. Besides glucose, free fatty acid can be used as an alternate source of energy, since fatty acid β-oxydation (FAO) can be used to generate ATP (adenosine triphosphate). The inhibition of FAO by blocking carnitine-palmitoyltransferase 1 (CPT1) in endothelial cells revealed that FAO was however mainly responsible for nucleotide synthesis. Accordingly, its inhibition reduced endothelial cell proliferation [33]. Furthermore, the inhibition of CPT1 reduced vessel formation in an ocular mouse model of angiogenesis, highlighting the anti-angiogenic potential of such an approach [33]. No doubt, targeting the endothelial metabolism is promising and needs to be investigated further. Nevertheless, similarly to growth factors inhibition, endothelial cells seem to have the ability to compensate for the inhibition of a specific metabolic pathway, and thus escape such therapies. Indeed, in vivo models have shown that angiogenesis is reduced but not inhibited. Hence, targeting the tumor endothelium by blocking endothelial cell metabolism might not provide long lasting effects.

Interestingly, immunological approaches to disrupt tumor vasculature were also successfully tested in pre-clinical studies [34,35]. Tumor endothelial cells represent a promising target, as they express in part markers that are distinct from normal endothelial cells. Most strategies used active immunization against VEGFR-2, and resulted in tumor growth inhibition [36,37,38]. In addition, T cells expressing a chimeric antigen receptor targeting tumor vasculature antigens were engineered and demonstrated a significant delay of tumor growth in pre-clinical studies [39,40]. However, as mentioned previously, tumor endothelial cells exhibit a remarkable heterogeneity, and hence targeting one antigen might not be sufficient to destroy the entire tumor vasculature. For instance, VEGFR-2 expression by endothelial cells varies considerably within the same tumor [41].

The emerging evidence demonstrate that tumor cells can further foster an angiogenic response via the secretion of extracellular vesicles such as exosomes and microvesicles [42]. These membrane enclosed particles released by cells can act locally and systemically [43,44]. Several reports have outlined that extracellular vesicles of tumor cells stimulate endothelial cell functions relevant to angiogenesis [45,46,47,48]. Accordingly, several pro-angiogenic factors including interleukin-6 (IL-6), interleukin-8 (IL-8), VEGF, or microRNA-29 (miR-29a) and microRNA-30a (miR-30a) have been detected in extracellular vesicles [49,50]. Hence, future studies will define whether therapeutic strategies targeting extracellular vesicles can block tumor angiogenesis.

It is further important to note that disrupting tumor endothelial cells has major consequences on the biology of tumors. Clinical studies using magnetic resonance imaging (MRI) have demonstrated that the responses to anti-angiogenic therapies include reduced perfusion, no changes in perfusion, and increased perfusion [51,52]. Hence, in the case of reduced perfusion, an abnormal tumor microenvironment is amplified by increased hypoxia and acidosis. The tumor hypoxic response is therefore further upregulated, resulting in major metabolic and phenotypic changes, such as increased invasion, tumor progression, and resistance to treatments [51]. In addition, hypoxia and acidosis strongly influence cells present in the tumor microenvironment; examples are the transformation of phenotype of resident macrophages into protumorigenic and immunosuppressive macrophages [53,54]. Furthermore, hypoxia and acidosis participate in tumor mediated immune evasion by reducing the cytolytic potential of immune effector cells [55,56]. Moreover, doses of anti-angiogenic treatments that increase tumor hypoxia frequently induce an immunosuppressive microenvironment. For example, the VEGFR tyrosine kinase inhibitor sorafenib increased the tumor infiltration of T regulatory cells, myeloid derived suppressor cells, and M2-type macrophages in a mouse model of liver cancer. It also upregulated programmed death-ligand 1 (PD-L1) expression by cancer cells, a ligand that negatively regulates the activity of T lymphocytes [57]. PD-L1 expression is in part regulated by hypoxia inducible factor-1α (HIF-1α), and therefore increases following anti-angiogenic treatments that induce hypoxia [58,59]. Hence, targeting the hypoxic adaptation of tumor cells in combination with anti-angiogenic therapies might improve the efficacy of anti-angiogenic therapies [60]. Several pre-clinical studies substantiate this hypothesis. For example, a knock-down of HIF-1α in combination with anti-angiogenic therapy reduced tumor growth in neuroblastoma xenografts [61]. Similarly, blocking carbonic anhydrase IX, an enzyme whose expression is increased by hypoxia and which regulates intratumoral pH, enhanced the anti-cancer efficacy of anti-angiogenic drugs [62,63]. Also, buffering intratumoral acidity with sodium bicarbonate in combination with anti-VEGF therapy provided stronger anti-cancer effects compared to anti-VEGF treatment alone [64]. Thus, in patients where the pro-hypoxic effect of anti-angiogenic drugs has been documented, the addition of therapies that target the hypoxic tumor response might substantially increase the anti-tumor benefits. Ongoing clinical trials are currently addressing this hypothesis in cancer patients [60].

## 3. Normalizing the Tumor Vasculature

Tumor blood vessels present many vascular abnormalities, such as increased permeability, dilation and tortuosity, reduced pericyte coverage, and irregular basement membranes, presumably due to an overstimulation by pro-angiogenic factors such as VEGF [65,66]. Consequently, tumor vessels function poorly, resulting in increased intra-tumoral fluid pressure and hypoxia, which further confer a resistance to chemo-, radio-, and immunotherapies [67]. The observation that the anti-VEGF agent bevacizumab provides survival benefits only when given with chemotherapy [10,11], and that patients whose tumor hypoxia decreased following treatment with anti-angiogenic treatments survive longer lead to a novel hypothesis on how anti-angiogenic drugs function. It was proposed that anti-angiogenic treatments could transiently normalize the dysfunctioning tumor vasculature, resulting in increased tumor blood perfusion and decreased tumor hypoxia, which in turn increases drug accessibility and reduces hypoxia-mediated treatment resistance [10,19]. Several pre-clinical studies support the vessel normalization hypothesis [68,69]. More importantly, vessel normalization by anti-angiogenic drugs was also observed in cancer patients. For instance, a single dose of bevacizumab was sufficient to induce vessel normalization in rectal carcinoma patients [70]. Similarly, normalized tumor vessels were noted in glioblastoma patients treated with cediranib, a pan-VEGF receptors inhibitor, and an improvement in overall survival correlated with the degree of normalization [71,72,73].

Besides increasing the delivery of chemotherapy and augmenting the efficacy of radiotherapy, normalizing tumor blood vessels benefits immunotherapy. As mentioned previously, tumor hypoxia and acidosis reduce the cytolytic activity of T lymphocytes [56]. Hypoxia further fosters an immunosuppressive tumor microenvironment by recruiting T regulatory cells and polarizing tumor associated macrophages to an M2 phenotype [53,74]. Hence, alleviating tumor hypoxia by vessel normalization represents a promising strategy to increase the efficacy of immunotherapies [75]. Consistent with this hypothesis, pre-clinical studies have demonstrated that treatments that induce tumor vessel normalization potentiate the anti-cancer efficacy of immunotherapies [75]. For example, low doses of an anti-VEGFR-2 blocker that increased tumor perfusion induced an immunocompetent tumor microenvironment characterized by CD8^+^ T lymphocytes and anti-tumorigenic M1 tumor associated macrophages infiltration [75]. Furthermore, it potentiated the effect of a cancer vaccine [76]. Similarly, low doses of tumor necrosis factor-α (TNF-α) normalized tumor blood vessels and improved adoptive T cell transfer therapy [77]. In addition, combined VEGF and angiopoietin-2 inhibition normalized tumor blood vessels, resulting in the perivascular accumulation of activated CD8^+^ T cells [78]. Furthermore, genetically induced vascular normalization potentiated anti-tumor immunity. In a pancreatic insulinoma mouse model, an ablation of regulator of G protein signaling 5 (RGS5) induced vascular normalization and improved adoptive T cell transfer therapy [79]. Likewise, vascular normalization induced by an overexpression of histidine-rich glycoprotein was associated with increased dendritic cell and CD8^+^ T cell tumor infiltration [80].

Interestingly, T lymphocytes, in particular CD4^+^ T_H_1 cells, actively participate in vessel normalization, highlighting the presence of a mutual regulatory loop [81]. Indeed, the depletion of CD4^+^ T lymphocytes decreases the coverage of tumor endothelial cells by pericytes. Furthermore, human tumors transplanted into immunodeficient mice exhibit areas of hypoxia that can be reduced following T_H_1 adoptive transfer [81]. Moreover, immune checkpoint inhibitor-mediated CD4^+^ lymphocytes’ activation increases vessel normalization. At the molecular level, interferon-γ and CD40L are both important signaling intermediates in this context [81]. Hence, a positive feedback loop exists where vessel normalization increases T lymphocyte recruitment and activation. In turn, the recruited CD4^+^ lymphocytes further increase vessel normalization.

**Figure 2:**
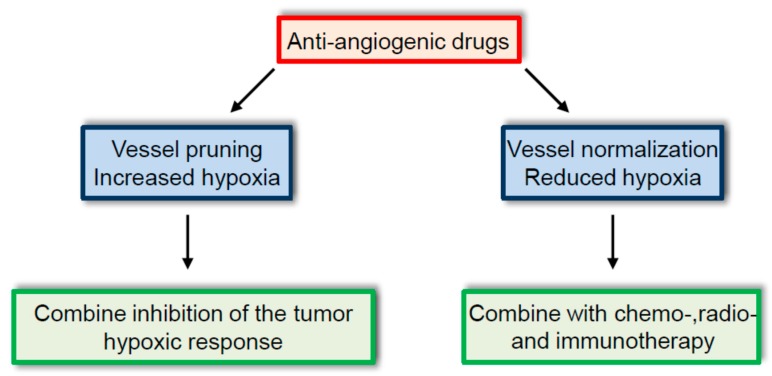
Consequences of current anti-angiogenic treatments. Anti-angiogenic therapies lead either to increase tumor hypoxia following tumor blood vessel destruction or reduced hypoxia by vessel normalization. Precise clinical monitoring of these effects will be important to further elaborate therapeutic strategies.

It is important to mention that tumor vessel normalization is not observed in all tumors treated with anti-angiogenic agents. As discussed previously, tumor blood vessels can be destroyed by anti-angiogenic drugs, but they can also be intrinsically resistant to anti-angiogenic therapies. Furthermore, the effect might change over time, as tumor vessel normalization might only be transient. In addition, due to the heterogeneity of tumor blood vessels, different effects of anti-angiogenic drugs are found in the same tumor. In the future, it will therefore be crucial to clearly identify the primary effect of these therapies in a given tumor, as this will have a major impact on the biology of the tumor. Indeed, in cases where anti-angiogenic drugs induce vessel normalization, chemo-, radio-, and immunotherapy will provide stronger benefits. In contrast, where anti-angiogenic drugs induce the tumor vessel pruning associated with increased tumor hypoxia, greater anti-tumor efficacy will be achieved by concomitantly blocking the tumor hypoxic response (Figure 2). Finally, resistant tumor blood vessels should encourage the discontinuation of anti-angiogenic treatments.

## 4. Shaping the Tumor Vasculature to Increase Anti-Tumor Immune Response

The tumor endothelium represents a barrier between the blood and the tumor. As such, it directs the selective trafficking of immune cells by the expression of adhesion molecules such as intercellular cell adhesion molecule 1 (ICAM-1), vascular cell adhesion molecule-1 (VCAM-1), and chemoattractants. In addition, it affects the activity of T lymphocytes by expressing major histocompatibility complex-I (MHC-I), MHC-II, and co-stimulatory molecules [12,14,82,83]. Hence, promoting endothelial cell functions that favor the immune response represents a therapeutic opportunity.

**Figure 3:**
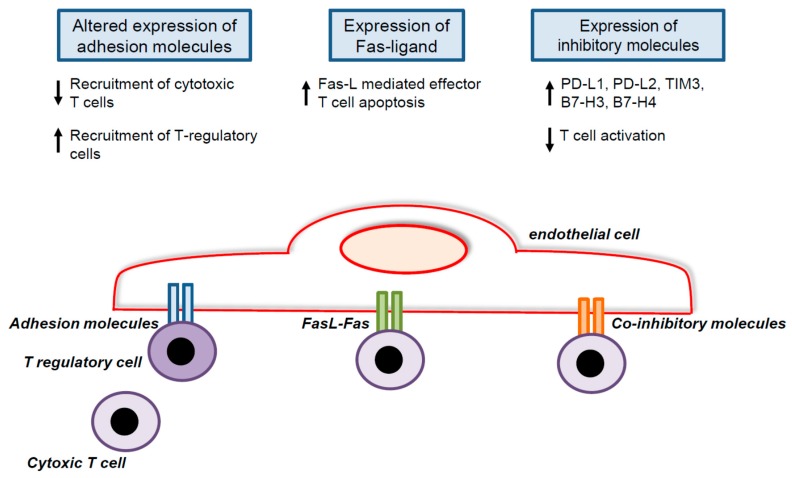
Mechanisms by which tumor endothelial cells affect host immune response to cancer. Tumor endothelial cells preferentially recruit T-regulatory cells in the tumor microenvironment. They promote effector T cell apoptosis in a FasL/Fas dependent manner. They reduce T cell activation by expressing co-inhibitory molecules. Up arrows signify increase, whereas down arrows signify decrease.

Quiescent endothelial cells usually become activated by inflammatory signals, resulting in the recruitment of leucocytes into inflamed tissues. In the context of cancer, these signals however mostly fail to induce the expression of the adhesion molecules necessary for leucocyte trafficking [84,85,86]. This lack of response, termed endothelial anergy, is induced in part by pro-angiogenic factors, including VEGF and basic fibroblast growth factor (bFGF), which contribute to the exclusion of immune cells from the tumor [87,88]. Conversely, anti-angiogenic therapies upregulate the expression of adhesion molecules by tumor endothelial cells [89,90]. Other factors implicated in tumor endothelial cell anergy include epidermal growth factor-like domain 7 and endothelin-1 through the endothelin B receptor [91,92]. Interestingly, in the case of endothelin-1 and VEGF, nitric oxide (NO) antagonists restore T cell adhesion by abrogating the deregulation of adhesion molecules [92,93]. In order to reduce anti-tumor immunity, tumor endothelial cells are able to upregulate adhesion molecules that favor the intra-tumoral infiltration of immunosuppressive cells. For instance, tumor endothelial cells promote the accumulation of T regulatory cells through the increased expression of common lymphatic endothelial and vascular endothelial receptor-1 (Figure 3) [94]. The preferential recruitment of T regulatory cells was also noted in human pancreatic carcinoma [95].

Several therapeutic approaches have been developed to reverse tumor endothelial cell anergy and thus favor the intra-tumoral recruitment of anti-tumor immune cells. As mentioned earlier, the use of vessel normalizing doses of anti-VEGF therapies increases T cell infiltration into tumors and augments the anti-cancer efficacy of immunotherapies [76]. Of note, blocking the endothelin B receptor is tested in clinics and seems to be well tolerated in cancer patients; the same is true for NO inhibitors [96,97]. A further approach uses the delivery of TNF-α, a major mediator of inflammation, to neo-angiogenic vessels. As the systemic administration of TNF-α has major side effects, alternative strategies to selectively target tumor vessels had to be developed [98]. This was achieved by fusing TNF-α with a peptide containing the CNGRC motif (NGR-TNF) sequence that interacts with CD13 on tumor vessels [99]. The administration of the NGR-TNF protein results in VCAM-1 and ICAM-2 upregulation on the tumor endothelium, favoring T cell trafficking [100]. NGR-TNF further increased the anti-cancer efficacy of vaccine and adoptive T cell transfer therapies [100]. Finally, the activation of CD137, a co-stimulatory molecule expressed by tumor vessels, also increased the expression of adhesion molecules by tumor endothelial cells. It further increased T cell homing into tumor tissues after an adoptive transfer which was inhibited by anti-ICAM-1 and anti-VCAM-1 blocking antibodies [101].

High endothelial venules (HEV) are post-capillary venules found in secondary lymphoid organs. They specifically promote the trafficking of naïve lymphocytes into lymphoid organs. Several tumors display blood vessels with HEV features, and the presence of HEV in tumors correlates with the presence of CD8^+^ cells and a favorable outcome [102,103]. Interestingly, recently the formation of HEV in tumors was induced by combined anti-VEGFR-2 and anti-PD-L1, which promoted antitumor immunity by recruiting more activated lymphocytes into the tumor [104]. Thus, therapeutic opportunity exists in inducing HEV formation in tumors to increase the recruitment of T lymphocytes in cancer tissues.

Besides recruiting blood vessels into cancers, tumor endothelial cells are able to influence the activity of T lymphocytes [12,13]. Indeed, endothelial cells express MHC class I and MHC class II molecules as well as co-stimulatory molecules [105]. In the context of cancer, the co-inhibitory molecule T cell immunoglobulin and mucin-domain containing-3 TIM3 was detected on lymphoma endothelium and contributed to immune evasion (Figure 3) [106]. Further co-inhibitory molecules, such as B7-H3 and B7-H4, were found on tumor blood vessels of various cancers, including cervical, endometrial, ovarian, and renal cell cancer. They correlated with a poor prognosis and in certain cases with reduced CD8^+^ T cell infiltration [107,108,109,110,111]. Furthermore, the expression of the immunosuppressive enzyme indoleamine 2,3-dioxygenase in tumor endothelial cells of renal cell carcinoma was detected [112]. Tumor endothelial cells are also able to secrete inhibitory molecules such as IL-6 or IL-10 [113,114]. Taken together, these observations demonstrate that tumor endothelial cells possess all of the machinery necessary to influence T cell activity. Future studies are needed to clarify the extent to which these mechanisms contribute to the immunosuppressive tumor microenvironment. Besides, pathways that regulate co-stimulatory or co-inhibitory molecule expression by tumor endothelial cells have the potential to be targeted and should hence be identified. For instance, the treatment of endothelial cells with the mTOR inhibitor rapamycin upregulated PD-L1 and PD-L2 expression on endothelial cells, and was associated with the reduced infiltration of effector T cells in an arterial allograft model [115].

Finally, tumor endothelial cells also have the ability to directly kill activated CD8^+^ T cells. Following stimulation with VEGF, IL-10, or prostaglandin E_2_, tumor endothelial cells express Fas ligand (FasL), which kills activated CD8^+^ T lymphocytes but not regulatory T cells (Figure 3) [116]. Accordingly, the inhibition of VEGF or prostaglandin E_2_ with aspirin blocked FasL expression by tumor blood vessels and resulted in increased CD8^+^ T cell infiltration and reduced tumor growth. Moreover, several cancer types characterized by FasL expression on tumor endothelial cells displayed reduced intra-tumoral CD8^+^ T cell infiltration [116]. Hence, the inhibition of FasL expression by tumor endothelial cells by simple means such as aspirin provides an interesting therapeutic approach and is being tested in clinical trials (NCT02659384).

## 5. Reinforcing the Endothelial Barrier to Prevent Metastasis

Metastasis is a complex procedure during which tumor cells disseminate to other parts of the body [117,118]. In particular, this presumes the entry of cancer cells into the bloodstream by disrupting the endothelial barrier in a process called intravasation [119]. Consequently, molecular changes that loosen the endothelial barrier favor cancer cell intravasation. In particular, the specific structural features of tumor endothelial cells, i.e., reduced pericyte coverage, leakiness, and weak interactions between adjacent endothelial cells facilitate intravasation. Accordingly, factors that promote the formation of immature tumor blood vessels, such as cyclooxygenase-2, promote cancer cell intravasation [120]. Conversely, therapies that induce vessel normalization decrease metastasis in animal models [121]. Additional molecular mechanisms that influence the ability of cancer cells to cross the endothelial barrier have been identified. For instance, a deficiency of endoglin, a co-receptor for transforming growth factor β (TGF-β), on the tumor vasculature causes a weakened endothelial cell barrier. This is associated with an increased tumor cell intravasation, presumably by the induction of an endothelial-to-mesenchymal transition [122]. Also, blood vessel-associated macrophages attract cancer cells and enhance intravasation by the secretion of epidermal growth factor and colony-stimulating factor-1 [123]. Furthermore, the repression of NOTCH signaling by the amino-terminal enhancer of split blocks cancer cell intravasation by inhibiting transendothelial migration of tumor cells [124]. Finally, cell division control protein 42 homolog (CDC42)-mediated β1 integrin expression also facilitates cancer cell interaction with endothelial cells and transendothelial migration [125].

Whereas experimental evidence show that reinforcing the endothelial barrier reduces tumor cell intravasation and metastasis, the clinical application of such therapies may be challenging. Of note, at the time of the initial diagnosis, cancer patients frequently exhibit undetected micrometastasis, hence strengthening the endothelial barrier might not be useful.

## 6. Modulating Angiocrine Signals Secreted by Tumor Endothelial Cells to Influence Tumor Growth and Response to Treatment

**Figure 4:**
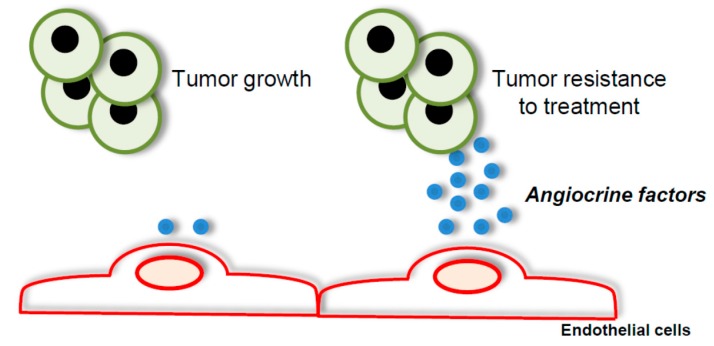
Angiocrine factors secreted by tumor endothelial cells promote tumor growth and resistance to anti-cancer treatment.

Tumor endothelial cells also influence tumor growth and response to treatments by secreting factors termed angiocrine factors (Figure 4) [126]. Hence, targeting angiocrine signaling mediated by tumor endothelial cells represents a therapeutic opportunity. Several of these angiocrine factors have been identified. For instance, Slit2, which is under the negative control of the endothelial receptor ephrin type-A receptor 2 (EphA2), suppresses tumor cell growth and motility [127]. Furthermore, the secretion of cytokines such as IL-1, IL-3, and IL-6, or the production of nitric oxide by tumor endothelial cells favors leukaemic cell proliferation [126,128]. Besides stimulating or suppressing tumor progression, angiocrine factors produced by endothelial cells were shown to modulate the response of tumor cells to anti-cancer therapies. In particular, the role of endothelial focal adhesion kinase (FAK) has been discussed in this process [129]. The specific loss of endothelial FAK sensitizes tumor cells to doxorubicin or irradiation. Mechanistically, doxorubicin and irradiation induce the secretion of cytokines by endothelial cells in an nuclear factor κ-light-chain-enhancer of activated B cells (NF-κB) dependent manner, including granulocyte-macrophage colony-stimulating factor (GM-CSF) and IL-6 that promote tumor resistance. In the absence of endothelial FAK, doxorubicin and irradiation fail to activate NF-κB in endothelial cells, resulting in a decreased production of cytokines and tumor cell sensitization to anti-cancer therapies [129]. A role in chemoresistance for IL-6 produced by endothelial cells has further been reported [130].

## 7. Conclusions

Targeting the tumor endothelium has evolved into an important modality in cancer therapy. Emerging evidence demonstrate that features of tumor blood vessels can be specifically targeted to generate distinct effects. The initial strategies aimed at preventing the formation of new blood vessels have provided limited benefits in clinical trials. The development of resistance mechanisms and increased tumor hypoxia contribute to this restricted efficacy. The ongoing clinical trials targeting tumor hypoxic response or resistance mechanisms will clarify whether the anti-cancer efficacy of a tumor blood vessel disrupting approach can be improved. Treatment modalities that lead to vessel normalization represent a promising tool to improve the efficacy of chemo-, radio-, and immunotherapy. They will have to be characterized in detail in order to identify the therapeutic windows for tumor blood vessel normalization. The approaches aiming to improve tumor endothelial cell-mediated lymphocyte recruitment and activation will have to be tested in clinical settings. Strategies to reinforce the endothelial barrier in order to reduce cancer cell dissemination exist; their practical applications need however to be closely considered. Finally, targeting angiocrine signals have shown anti-tumor efficacy in experimental models that will need to be translated in patients. Since the role of tumor endothelium in cancer is complex, any additional features of tumor endothelial cells that influence tumor progression and that can be therapeutically targeted will be further identified in the near future.
